# Ethics of disclosure of onset‐predictive biomarker test results for genetic frontotemporal dementia in the research context

**DOI:** 10.1002/dad2.70133

**Published:** 2025-06-11

**Authors:** Charlotte H. Graafland, Laura Donker Kaat, Edo Richard, John C. van Swieten, Harro Seelaar, Eline M. Bunnik

**Affiliations:** ^1^ Department of Public Health Program Medical Ethics, Philosophy and History of Medicine Erasmus University Medical Center Rotterdam the Netherlands; ^2^ Department of Clinical Genetics Erasmus University Medical Center Rotterdam the Netherlands; ^3^ Department of Neurology Donders Institute for Brain Cognition and Behaviour, Radboud University Medical Center Nijmegen the Netherlands; ^4^ Department of Public & Occupational Health Amsterdam University Medical Center, University of Amsterdam Amsterdam the Netherlands; ^5^ Department of Neurology and Alzheimer Center Erasmus University Medical Center Rotterdam the Netherlands

**Keywords:** biomarker, clinical trial, disclosure, ethics, frontotemporal dementia, informed consent, onset prediction, recruitment

## Abstract

Onset‐predictive biomarker tests (OPBTs) for genetic frontotemporal dementia (FTD) may predict symptom onset in coming years. OPBT results could be used as inclusion criterion for clinical trials for FTD, but this requires disclosure of OPBT results to potential participants for informed consent. This creates a dilemma, as disclosure may be psychologically burdensome. Yet, individuals at risk of FTD may value OPBTs to relieve uncertainty and support planning for the future. This article provides an overview of considerations regarding disclosure using four themes (actionability, respect for autonomy and informed consent, psychological impact, social and societal impact), based on literature on return of individual research results and biomarker disclosure in Alzheimer's disease. Based on this, we argue that clinical validity and context of use are important considerations, and suggest that (1) counseling facilitates informed decision making, (2) clinical and psychological follow‐up provides necessary support, and (3) impacts may be monitored in a pilot study.

**Highlights:**

- Ethical considerations regarding disclosure of onset‐predictive biomarker test (OPBT) results include actionability, respect for autonomy and informed consent, psychological impact, and social and societal impact.
- The weight of each consideration depends heavily on the clinical validity of the OPBT results and the context of use.
- OPBT result disclosure to individuals at risk of genetic frontotemporal dementia (FTD) for clinical trial recruitment seems ethically acceptable.
- We suggest embedding OPBT results disclosure in counseling, follow‐up, and a pilot study on impacts of OPBT results disclosure.

## 1 INTRODUCTION

Frontotemporal dementia (FTD) is characterized by progressive behavioral, language, and/or motor symptoms, such as disinhibition, apathy, emotional blunting, loss of empathy, or different types of aphasia.^1^ FTD has an autosomal dominant inheritance pattern in 20% to 30% of cases,^1^ so that children of genetic FTD patients have a 50% risk of inheriting the pathogenic variant. Many potential carriers do not pursue presymptomatic genetic testing for various reasons, including the current lack of disease‐modifying treatments.^2^

Clinical trials are increasingly being conducted for genetic FTD, of which some aim to prevent or delay neurodegeneration by intervening early in the disease process, prior to symptom onset. The presumed “window of opportunity” for treatment is at the start of the disease process, when irreversible damage has not yet or minimally occurred. Therefore, researchers aim to recruit participants who (1) carry a pathogenic variant causing FTD and (2) are expected to develop symptoms in the coming years. However, the age of onset in carriers is highly variable even within families, ranging from their 40s to 80s.^1^, ^3^ This variability hampers selection of clinical trial participants approaching symptom onset based solely on age.

The selection process could be supported by “onset‐predictive biomarker tests” (OPBTs) that indicate disease activity and forewarn symptom onset. An ongoing clinical trial for latozinemab used serum neurofilament light chain (NfL) as an inclusion criterion in presymptomatic *GRN* carriers.^4^ Serum NfL levels are a non‐specific biomarker of neuronal injury and are elevated in several neurological disorders, including amyotrophic lateral sclerosis (ALS)^5^ and multiple sclerosis (MS).^6^ In genetic FTD, serum NfL and other (combinations of) OPBTs correlate well with symptom onset on the group level, but are not suitable to predict onset in individuals.^7^, ^8^, ^9^ In the near future, OPBTs with higher predictive value may be developed to aid clinical trial recruitment of carriers who are approaching symptom onset. Promising examples include fluid biomarkers such as cryptic exons^10^ or neuronal pentraxin‐2,^11^ neuropsychological tests, and magnetic resonance imaging biomarkers.

Longitudinal cohort studies of genetic FTD such as the Genetic Frontotemporal Dementia Initiative (GENFI) and the ARTFL‐LEFFTDS Longitudinal Frontotemporal Lobar Degeneration (ALLFTD) study may serve as “trial‐ready” cohorts. Both participants with known carrier status or 50% risk may undergo OPBTs to assess whether they are eligible or “ready” to participate in a clinical trial, in terms of meeting (biologically defined) inclusion criteria.^12^ If the OPBT is non‐specific for FTD, individuals at 50% risk would also need to undergo genetic testing to be eligible for clinical trials in genetic FTD. Ethical guidelines for interventional study recruitment recommend that eligible participants should be enrolled for clinical trial participation using “transparent enrollment”.^13^, ^14^ This means that, as part of the informed consent process, they should be informed that their positive OPBT result is the reason for their eligibility. Disclosing these individual research results in the process of enrollment (“disclosure by enrollment”^14^, ^15^) raises ethical concerns about adverse impacts of disclosure on research participants.

The impact of disclosing OPBT results to research participants has not yet been studied. OPBT results providing information about time to onset may be more burdensome than presymptomatic genetic testing, especially in individuals who simultaneously learn about their carrier status and the imminence of disease onset. A concern is that this may lead to increases in self‐monitoring for symptoms (“hypervigilance”) and anxiety about onset. Yet, known carriers and individuals at 50% risk of FTD often experience hypervigilance and psychological distress in their daily lives due to uncertainty about age of onset.^16^, ^17^ For them, onset‐predictive information and the opportunity to participate in clinical trials to contribute to treatment development may be valuable.

As the benefits and risks of OPBT result disclosure are unclear, researchers grapple with the ethical acceptability of OPBT result disclosure in the context of clinical trial recruitment. This issue has urgent practical importance, as more clinical trials for genetic FTD may soon use OPBTs as an inclusion criterion. This article aims to provide an overview of ethical considerations regarding OPBT result disclosure in the context of clinical trial recruitment in longitudinal cohort studies. This overview may stimulate the academic discussion on this topic and help research groups determine whether to disclose OPBT results to study participants. We argue that clinical validity and context of use are important factors when deciding about disclosure of OPBT results in research, and suggest the implementation of counseling and follow‐up with disclosure of OPBT results.

## 2 METHODS

The considerations are drawn from two bodies of literature: the disclosure of Alzheimer's disease (AD) biomarker results and the return of individual (genomic) research results. The literature on AD biomarker disclosure mainly discusses two types: apolipoprotein E (*APOE*) genetic status and amyloid positron emission tomography (PET) results. These differ from OPBTs for genetic FTD in three ways: (1) they have predictive value for late‐onset dementia in the general population rather than young‐onset dementia in carriers of a highly penetrant mutation, (2) they convey a relatively low risk of developing AD dementia rather than a high risk of quickly developing FTD, and (3) they convey a risk of developing dementia at some point, instead of predicting time to onset of a few years.

Given that both amyloid PET and FTD biomarkers provide information about current brain pathology and are indicative of dementia risk, some ethical considerations regarding AD biomarker disclosure are adaptable to OPBTs. The broader literature on the return of individual (genomic) research results is also relevant, as it extensively addresses arguments regarding disclosure of different types of individual research results. In addition to these two bodies of literature, we also included considerations based on our recent qualitative interview study in Dutch carriers and individuals at 50% risk of genetic FTD, some of whom were longitudinal cohort study participants, on their perspectives on OPBTs.^18^

We inductively organized the considerations found in the literature into four themes: actionability, respect for autonomy and informed consent, psychological impact, and social and societal impact.

## 3 FOUR THEMES OF ETHICAL CONSIDERATIONS

### 3.1 Actionability

An important normative consideration noted in the literature on returning individual research results is the result's “actionability”. Actionability refers to the extent to which the test result is relevant for decision making and action taking and comes in two forms.

The first is clinical utility, defined as “evidence of improved measurable clinical outcomes, and [the test's] usefulness and added value to patient management decision‐making compared with current management without (…) testing”.^19^ OPBTs for FTD have limited clinical utility, because there are currently no approved disease‐modifying therapies or evidence‐based prevention strategies for FTD.

The second is personal utility, the test result's “potential to effect change on a (non‐medical) personal level”.^20^ In the field of AD biomarkers, personal utility is increasingly seen as sufficient justification to return individual research results, even in the absence of clinical utility.^21^, ^22^ Individuals at risk of genetic FTD may attribute personal utility to OPBT, as a prediction of time to onset allows them to make choices in three domains: health promotion (changing lifestyle, participating in a clinical trial), life planning (working less or retiring, spending time on hobbies and with loved ones) and arranging future affairs (care and professional help, finances, advance care directives).^18^ A survey study among British longitudinal genetic FTD cohort participants found that 85% were willing to receive NfL results with invitations to participate in clinical trials.^23^ In our interview study, we found that some individuals at 50% risk view OPBT results as more actionable than genetic status alone, and would prefer to receive OPBT and genetic results simultaneously—rather than receiving their genetic test result years or decades before onset.^18^ The question remains whether individuals at risk of genetic FTD will actually make the changes they expect to make if they receive OPBT results.

Importantly, the actionability of OPBT results depends on the clinical validity of the test. If the positive or negative predictive values are low, making decisions based on this information may not be epistemically justified.^20^, ^24^ Whether the diagnostic accuracy is sufficient depends on the context and purpose of the test, and the relative importance of specificity and sensitivity.^25^ For example, the choice to participate in a clinical trial associated with limited burdens may be justified by an OPBT result with a lower clinical validity, while radical personal choices regarding work and finances may require a higher level of clinical validity. Individuals at risk of genetic FTD differ in their perspectives on what the positive and negative predictive value should be for them to consider making personal choices based on OPBT results. When asked, the minimum mentioned was 70%, while other interview participants set the bar at 90% to 95%.^18^ In the academic community, a diagnostic test with an area under the curve (AUC) of > 0.8 is usually considered acceptable.^25^ What cut‐off values should be used for an optimal balance between specificity and sensitivity is yet to be determined, and may depend on the context of use.

### 3.2 Respect for autonomy and informed consent

Two prominent ethical considerations are the right to access information and the right not to know. The former entails that individuals have a right to access information about their health.^15^, ^26^ Proponents of AD biomarker disclosure have argued that withholding potentially useful prognostic biomarker results from research participants is paternalistic and disrespects their autonomy.^21^, ^22^ In addition, some argue that researchers show respect to participants by returning individual research results, expressing reciprocity for their participation in research.^26^, ^27^, ^28^ Reciprocity may motivate potential participants to enroll in the study or participate longer, which improves recruitment and retention.^14^, ^21^, ^29^, ^30^

The right not to know implies that individuals should be able to refuse information they do not wish to receive. The right serves two purposes: it respects the individual's autonomy to decide what information they do not wish to know, and it prevents psychological harm from learning unwanted information.^31^ This right is preserved in current longitudinal FTD studies, as participants are not required to be informed of their genetic status.^32^ Unwanted disclosure of OPBT results by enrollment can be avoided by asking longitudinal cohort study participants for informed consent for being monitored using OPBT and disclosure of results.

Informed consent entails empowering a potential participant to make a voluntary, well‐considered and well‐informed decision about participation. The participant has to be provided with and must understand all relevant information. But research participants may have difficulty understanding the meaning of OPBT, especially those with low health literacy and/or numeracy. In the field of AD biomarkers, a concern is that participants may consent to receiving results without sufficiently understanding the potential implications,^22^, ^33^ and may take inappropriate actions based on results.^34^ Applying this to OPBTs, understanding the prediction of time to onset—accompanied by ranges, confidence intervals, and positive and negative predictive values—is likely to be equally complex as understanding AD biomarker results. In addition, if the OPBT is not predictive in individuals, it may be hard to explain why the OPBT is used for trial recruitment. Thorough counseling and effective risk communication will help cohort participants make an informed decision about disclosure. Even then, some potential participants may not be able to fully comprehend all the relevant information and may perceive a positive OPBT result as an almost certain prediction of time to onset.

Another concern is whether it is acceptable to require participants to be informed of their biomarker results to be eligible for clinical trials. On the one hand, the requirement is not coercive, because study participation is not an excessive reward due to clinical equipoise.^30^ On the other hand, individuals may have therapeutic misconceptions about the efficacy of investigational compounds, (falsely) believing that they will benefit from participation. Because their access to the trial depends on willingness to undergo biomarker testing, they may accept disclosure when they might otherwise have refused it.^35^, ^36^ FTD research participants may have similar therapeutic misconceptions. The uncertainty regarding the efficacy of the investigational drug and the placebo‐controlled design should be carefully explained during the informed consent process.

### 3.3 Psychological impact

The potential psychological burden of receiving OPBT results may interfere with the ethical principle of non‐maleficence, which entails that researchers should avoid harming participants when possible. In the absence of empirical data, it is difficult to estimate what the psychological impacts of OPBT result disclosure will be. The appropriateness of these effects will depend on the clinical validity of the OPBT and the individual's correct understanding of the OPBT result's significance.

The interview study in carriers and individuals at 50% risk of FTD found that they expected (1) test‐related distress and (2) psychological impacts from OPBT disclosure.^18^ Test‐related distress surrounding the testing procedure might occur especially in individuals at 50% risk, as regular OPB testing is comparable to repeatedly undergoing indirect genetic testing. Furthermore, if the result is inconclusive, the period of uncertainty until retesting may be experienced as distressing.

Disclosure of OPBT results may potentially have six negative psychological effects. The first three were described in the literature and/or mentioned in the interview study. First, positive OPBT results can lead to emotional burden from being confronted with news that symptom onset may occur soon.^18^ In individuals at 50% risk, this information would be combined with learning they are a carrier of genetic FTD, which could potentially have serious adverse effects. Second, results can negatively affect self‐image, causing the individual to feel like “a patient,” and thereby inducing or increasing hypervigilance.^18^, ^35^, ^37^, ^38^, ^39^ Third, there is evidence of a nocebo effect occurring after AD biomarker disclosure: individuals aware of their genetic susceptibility to AD scored lower on neuropsychological tests than those who were unaware of the same genetic susceptibility.^40^ We hypothesize that OPBT result disclosure may cause a similar effect.

The second three negative effects are hypothesized based on our experience. First, we believe that results with low clinical validity (e.g., false positives, false negatives, inconclusive results, positive results of a non‐specific OPBT combined with negative genetic results) may cause confusion, lead to regrets if used for radical decision making, inflict psychological harm when onset occurs unexpectedly,^18^ or lead individuals at 50% risk to feel forced to undergo genetic testing to clarify the result. Second, (repeatedly) receiving a negative OPBT result may lead individuals at 50% risk to (sometimes incorrectly) assume they do not carry the pathogenic mutation. Finally, individuals receiving negative OPBT results might experience survivor's guilt toward family members who are affected or receive positive OPBT results, as has been reported for negative genetic results in Huntington's disease.^41^

Disclosure of OPBT results may also have three positive psychological effects.^18^ First, disclosure of a positive OPBT result may give individuals a sense of control by providing the opportunity to prepare for onset. This positive effect may offset potential negative impacts of OPBT results, also in individuals at 50% risk of FTD, by providing them with the opportunity to prepare for onset. In the interview study, individuals at 50% risk expected that this benefit of positive OPBT results would outweigh the potential negative psychological effects.^18^ Second, OPBT disclosure may relieve uncertainty about time to onset. It may help carriers and caregivers to correctly interpret behavioral changes as resulting from the disease and to anticipate disease progression, rather than remaining in doubt whether the disease has started. Third, if the negative predictive value of the test is high, a negative OPBT result may reassure individuals at risk that onset is not expected in the near future. This may relieve uncertainty and potentially reduce hypervigilant behaviors.^18^

In sum, the psychological effects of disclosure of OPBT results may be diverse and remain uncertain (Table 1). Negative effects of disclosure of positive OPBT results to individuals at 50% risk could be serious, but could also be alleviated by the perceived sense of control and opportunity to prepare for onset.

**TABLE 1:** Overview of potential positive and negative psychological impacts of OPBT results.

OPBT result	Potential positive effects	Potential negative effects
Positive	‐ More certainty about onset ‐ Interpret first symptoms correctly ‐ Sense of control from preparation	‐ Psychological burden from approaching onset ‐ Negative self‐image as “patient” ‐ Increased hypervigilance ‐ Nocebo effect
Negative	‐ Relief that onset is not expected ‐ Reduced hypervigilance ‐ Allows individuals at 50% risk to postpone genetic testing	‐ Incorrect assumptions about next OPBT result or being a non‐carrier ‐ Survivor's guilt toward affected family members
Inconclusive or low clinical validity		‐ More uncertainty about onset than before testing for prolonged period ‐ Feeling forced to undergo genetic testing to clarify result
False positive		‐ Making life choices based on incorrect expectation of onset
False negative		‐ Onset is unexpected ‐ Not recognizing first symptoms because of negative OPBT result
Positive with negative genetic result	‐ Relief about non‐carriership	‐ Confusion about positive OPBT result

Abbreviation: OPBT, onset‐predictive biomarker test.

### 3.4 Social and societal impact

Positive OPBT results may affect social interactions between the person at risk and their social environment. In the direct social circle, positive OPBT results may negatively impact family or friendship dynamics. Literature on AD biomarker disclosure points out that family members or friends may increase surveillance of the person at risk after a positive biomarker result,^33^, ^37^, ^38^, ^42^ may second‐guess their decision‐making agency,^42^ or make pre‐emptive changes in the activities the person at risk is free to perform (e.g., take over financial affairs).^42^, ^43^, ^44^ Similar effects may occur after the disclosure of OPBT results in FTD, potentially leading to stigmatized interpretations of the affected individual's actions as disease‐related behaviors. This may lead to unnecessary and undue restrictions of their freedom, even if the disease is not manifest and there are no safety risks that might otherwise justify paternalistic interventions. In addition, the feeling of being watched and second‐guessed may have negative psychological effects on the person with positive OPBT results.

On the other hand, positive OPBT results may also have positive effects on family and friendship dynamics by helping to correctly interpret initial subtle behavioral symptoms as disease consequences rather than deliberate actions of the person affected. In this way, relationships with the social environment may be preserved and social support and care may be organized more quickly.^33^, ^45^ Thus, these effects may support the safety and well‐being of the person receiving positive OPBT results.

Positive OPBT results may also affect family members. First, a pre‐emptive role change to “pre‐caregiver” may occur, as has been described in partners of individuals with a positive AD biomarker result.^35^, ^37^, ^46^, ^47^ The positive OPBT result may signal to family members that they must soon take up the role of informal caregiver. Second, positive OPBT results in individuals at 50% have implications for the genetic risk of family members if a pathogenic variant is detected.

At the societal level, the development of biomarkers for dementia fits into a broader trend to conceptualize diseases causing dementia as a continuum that starts with a “preclinical” stage in which only biological pathology is present and symptoms are absent.^12^, ^48^ This reconceptualization of dementia may turn asymptomatic individuals with positive biomarker results into “patients‐in‐waiting”, who are characterized as having a disease on its way to becoming manifest.^38^ The risk of stigmatization based on biomarker results is frequently mentioned in the literature on AD biomarkers. Biomarker results may lead to loss of friends or social standing in anticipation of cognitive decline ^42^ ^.^ ^43^, ^47^ or lead to discrimination at work when employers are informed; for example, firing the person at risk or assigning different tasks.^22^, ^35^, ^36^, ^37^, ^45^, ^49^ Alternatively, in some countries such as the United States, biomarker research results could enter a person's medical record and be used for underwriting purposes by insurance providers.^49^ A similar change in perceived health status may occur for individuals who have received positive OPBT results in absence of symptoms, turning them into patients‐in‐waiting.

## 4 DISCUSSION

In this article, we provide an overview of ethical considerations regarding the disclosure of OPBT results in FTD research (Table 2). In summary, disclosure appears to be in line with the wishes of participants, who are willing or even eager to receive OPBT results in a hypothetical scenario.^18^ The results may have personal utility both by enabling individuals to enroll in clinical trials and by allowing life planning in various domains (e.g., work, care arrangements, leisure). Still, actionability and willingness to receive results also depends on the clinical validity of the test result.^18^ Individuals unwilling to receive OPBT results can be protected by asking for informed consent, to avoid paternalism and unwanted disclosure and show reciprocity to research participants.^28^ At the same time, obtaining informed consent may be challenging due to the highly complex nature of biomarker information. Furthermore, the potential psychological and societal impacts from disclosure of OPBT results are unpredictable and are a reason to proceed with caution.

**TABLE 2:** Overview of ethical considerations regarding disclosure of OPBT results.

Theme	Considerations
Actionability	No clinical utility in absence of disease‐modifying treatments
	Carriers and individuals at 50% risk perceive personal utility from OPBT results
	Apparent high willingness to receive OPBT results
	Utility is conditioned on a sufficient level of clinical validity
Respect for autonomy and informed consent	Participants have a right to access to their health information
	Withholding health information is paternalistic
	Disclosure is a form of reciprocity toward research participants
	Right not to know can be preserved by asking for informed consent prior to disclosure
	Information about OPBT is complex and may hinder informed consent due to a research participant's misunderstandings
	Participants may have therapeutic misconceptions about clinical trial participation that lead them to accept disclosure of OPBT results
Psychological impact	Undergoing OPBT regularly may cause test‐related stress
	Disclosure of OPBT results may have negative psychological effects such as emotional burden, negative self‐image and hypervigilance, a nocebo effect, and harm from false results
	Disclosure of OPBT results may have positive psychological effects such as relief about uncertainty of symptom onset, opportunity to prepare for onset, and relief after a negative result
	There is no empirical data available on the actual psychological effects of OPBT disclosure
Social and societal impact	Positive OPBT results may have negative effects on the family such as pre‐emptive role changes of family members, implications for genetic risk of family members, and increased surveillance of the OPBT‐positive individual
	Positive OPBT results may have positive effects on family dynamics, by helping explain initial FTD symptoms and organizing care sooner
	There is a risk of stigmatization based on OPBT results, affecting social standing and employment or insurance

Abbreviations: FTD, frontotemporal dementia; OPBT, onset‐predictive biomarker test.

These ethical considerations regarding disclosure of OPBT results must be viewed in light of the OPBT's clinical validity and its context of use. If the clinical validity is low and OPBT is used for the purpose of clinical trial recruitment, one of the main ethical issues is informed consent. It will be challenging to explain to participants that OPBT is being used for clinical trial recruitment while its clinical validity is low. This is complex information that complicates informed consent, especially for those with low health literacy or numeracy. In this context, researchers should be most concerned about potential adverse consequences of misunderstanding and overestimation of OPBT test results by research participants, such as rash decision‐making or disproportionate psychological burdens. In contrast, future OPBTs with a high clinical validity will lead to a different set of ethical issues, as disclosure may have significant medical, practical, psychological, social, and societal implications for carriers or those at 50% risk.

In addition to these considerations, research groups deciding whether to disclose OPBT results to research participants should consider local regulations and guidelines, as well as any existing agreements with cohort participants. Specific contextual factors of the study and characteristics of the research population may impact the magnitude of risks and benefits, and the weight of each consideration. For example, the current frequency of and type of tests conducted with longitudinal cohort study visits may impact the level of test‐related stress experienced. Participants in studies with less frequent visits may experience undergoing OPBTs more regularly as significantly more burdensome. Another example is that the proportion of carriers and individuals at 50% risk in the study population may affect willingness of participants to receive OPBT results.

Overall, the use of OPBTs for clinical trial recruitment in a transparent enrollment design may be ethically acceptable if the concerns about informed consent are accounted for in the study set‐up. Initially, only known carriers were screened for clinical trial eligibility for latozinemab using OPBT. Yet, there are two reasons to consider including individuals at 50% risk in OPBT before genetic testing. First, in this way individuals at 50% discover they are carriers only if they have a positive OPBT result and are eligible for trial participation. This eliminates the possibility that individuals at 50% risk regret undergoing genetic testing for the purpose of clinical trial recruitment if they are not eligible for study participation due to a negative OPBT result. Second, this expands the pool of potential clinical trial participants, as individuals at 50% risk can also be included, which increases the feasibility of running clinical trials for rare diseases like genetic FTD. Of course, there is a potential for serious negative psychological impacts of OPBT disclosure, especially in individuals at 50% risk. Yet, our study suggests that these individuals are willing to undergo OPBT and expect that they would be able to cope with simultaneous disclosure of OPBT results and genetic results, partially because of the perceived burdens of missing the opportunity to prepare for symptom onset.^18^ As long as there is no evidence on negative psychological impacts of OPBT results, it might be paternalistic to restrict access to OPBT for willing individuals at 50% risk. We therefore propose to offer OPBT to individuals at 50% risk, pending further evidence.

We make three suggestions to support an ethically responsible disclosure process should OPBT be used for clinical trial recruitment in the near future: (1) OPBT is accompanied by a pre‐test counseling process with informed consent; (2) there is post‐test clinical and psychological follow‐up, even if participants are not enrolled in the clinical trial; (3) OPBT results are initially only disclosed in a pilot study, so as to gather empirical evidence on the impacts of disclosure and make necessary adjustments to the disclosure process.

First, participants may be supported to make informed decisions about OPBT disclosure in a thorough counseling process. This is currently the recommended approach for genetic testing and biomarker testing in neurodegenerative diseases. This counseling should be carefully designed to effectively communicate all information relevant to decision‐making and support individual decision‐making processes. One important aspect to discuss in counseling is the predictive value of the OPBT for individuals. Different versions may be tailored to the specific target group, distinguishing between known carriers and individuals at 50% risk. The designs can be informed by existing protocols for disclosure of AD biomarker results. Counseling regarding disclosure of OPBT results may be offered to all study participants, which will require a significant time investment by study personnel.^26^, ^30^ This suggestion therefore presupposes that the necessary study budget and personnel are available to facilitate this.

Second, we suggest offering clinical and psychological follow‐up to individuals receiving OPBT results, specifically those receiving positive results. The purpose of psychological follow‐up is to help participants cope with positive results over time, should this be necessary. The purpose of clinical follow‐up is two‐fold: on the one hand, the participant is monitored for progression to the symptomatic stage of disease using neuropsychological testing, so that a timely diagnosis can be made and appropriate care provided. On the other hand, especially if the clinical validity of the OPBT is low or the OPBT is non‐specific, repetition of the OPBT over time can aid the interpretation of the previous result and support or refute the prediction that onset will occur soon. Both types of follow‐up may be offered to any participant who has received an OPBT result, even if not enrolled in the clinical trial.

Third, we suggest initially limiting disclosure of OPBT results to a small group in a pilot study. The aim would be to gather initial empirical evidence on the psychological effects of OPBT disclosure on individuals at risk of genetic FTD and their family members. This would allow an assessment of the impact of OPBT results before exposing a larger group to the risks of OPBT disclosure. In our view, it seems plausible that disclosure of OPBT results will lead to negative psychological impacts. Yet, this is not necessarily an argument not to disclose OPBT results. Participants who are informed of the potential negative psychological effects of disclosure may decide that these burdens do not outweigh the opportunity to participate in a clinical trial or prepare for onset. However, if disclosure of OPBT results is found to have significant negative psychological impacts, this may indicate that more extensive follow‐up and psychological support should be offered to help carriers cope. If the initial empirical evidence shows that understanding of the meaning of OPBT results and the psychological and social impacts are acceptable, a larger group can be invited to undergo OPBTs. This group may also be followed to assess psychological impacts, to expand on the first empirical evidence gathered in the pilot study.

To implement these suggestions, there is need for guidance on the process of disclosure of OPBT results, for example, regarding the contents of pre‐test counseling and post‐disclosure follow‐up. Furthermore, future research should explore how the ethical considerations listed in this article change with the context of use and clinical validity of the OPBT. For example, new research directions could be to explore under what conditions OPBTs might be offered in the clinical context, or how the development of disease‐modifying treatments could change these considerations.

In conclusion, there are many ethical considerations surrounding disclosure of OPBT results to individuals at risk of genetic FTD to take into account. Overall, disclosure of OPBT results seems ethically acceptable. By disclosing OPBT results in their study, researchers enter new territory regarding the type of information available to individuals at risk of genetic FTD. Disclosure of biomarkers in other neurodegenerative diseases, such as Huntington's disease, may quickly follow.^50^ Ethical guidance of disclosure of OPBT results is necessary to ensure these developments support rather than harm the well‐being of individuals at risk of genetic FTD.

## AUTHOR CONTRIBUTIONS

Harro Seelaar, John C. van Swieten, and Eline M. Bunnik conceived of the idea for the research and designed the study approach. Charlotte H. Graafland conducted the literature search and made an overview of all the considerations from the literature and analyzed the application to OPBTs in FTD together with Eline M. Bunnik. Harro Seelaar, John C. van Swieten, Edo Richard, and Laura Donker Kaat contributed to the overview of considerations in an early stage. Charlotte H. Graafland and Eline M. Bunnik drafted the article. All authors substantively revised and commented on the draft, and approve of the submitted version. All authors agree to be personally accountable for all aspects of the work.

## CONFLICT OF INTEREST STATEMENT

The authors declare no conflicts of interest. Author disclosures are available in the supporting information.

## CONSENT STATEMENT

No consent was necessary for this study, as no human subject data were collected.

## Supporting information



Supporting Information
